# Predictors of in-hospital case-fatality in patients with stroke at the National Hospital of the Kyrgyz Republic: a retrospective cohort study

**DOI:** 10.1186/s12883-026-05017-x

**Published:** 2026-05-29

**Authors:** Hyejin Choi, Jaewook Choi, Chubak Maksatbekov, Bakyt Tologonov, Kyunghee Kim

**Affiliations:** 1 Department of Public Health, Graduate School, Korea University, 73 Goryeodae-ro, Seongbuk-gu, Seoul, 02841 South Korea; 2 Department of Preventive Medicine, College of Medicine, Korea University, 73 Goryeodae-ro, Seongbuk-gu, Seoul, 02841 South Korea; 3 Institute for Environmental Health, Korea University, 73 Goryeodae-ro, Seongbuk-gu, Seoul, 02841 South Korea; 4 National Hospital of the Kyrgyz Republic, Togolok Moldo 1, Bishkek, 720040 Kyrgyz Republic

**Keywords:** Stroke, Hospital mortality, Asia, central, Kyrgyz Republic, Tertiary care centers, Risk factors, Retrospective studies, Sustainable development goals

## Abstract

**Background:**

Stroke-related mortality remains disproportionately high in Central Asia, with Kyrgyz Republic among the most affected countries. However, evidence on predictors of in-hospital death in this setting remains limited, particularly for comparative analyses of ischemic and hemorrhagic stroke. Identifying context-specific determinants of in-hospital death is essential for informing targeted strategies to improve acute stroke care and outcomes in resource-constrained health systems.

**Methods:**

This retrospective cohort study included 1,259 patients with stroke (1,033 ischemic and 226 hemorrhagic) admitted in 2024 to the Neurology Department No. 2 of the National Hospital of the Kyrgyz Republic. Sociodemographic, lifestyle, medical and admission-related characteristics were extracted. Multivariable logistic regression was applied separately to ischemic and hemorrhagic stroke to identify independent predictors of in-hospital case-fatality.

**Results:**

In-hospital case-fatality was 9.6% for ischemic and 21.7% for hemorrhagic stroke. In ischemic stroke, high Modified Early Warning Score (MEWS) (aOR = 75.1, 95% CI 29.18–193.21) and medium MEWS (aOR = 12.5, 95% CI 6.54–23.95) were strongest predictors of in-hospital case-fatality. Higher risk was noted among Bishkek residents (aOR = 4.2, 95% CI 1.72–9.94), those with prior myocardial infarction (aOR = 2.1, 95% CI 1.05–4.18) and ambulance admissions (aOR = 2.9, 95% CI 1.16–6.97). Overweight (aOR = 0.5, 95% CI 0.25–0.98) and hyperlipidemia (aOR = 0.5, 95% CI 0.26–0.84) were inversely associated with in-hospital case-fatality. In hemorrhagic stroke, high (aOR = 51.3, 95% CI 12.73–206.73) and medium (aOR = 7.5, 95% CI 2.23–25.40) MEWS, female sex (aOR = 2.8, 95% CI 1.09–7.36) and prior myocardial infarction (aOR = 5.94, 95% CI 1.45–24.29) increased case-fatality.

**Conclusion:**

This study provides early real-world, hospital-based evidence on predictors of in-hospital case-fatality among stroke patients in the Kyrgyz Republic. Early physiological risk stratification and targeted interventions for high-risk groups could improve outcomes and guide stroke care in low- and middle-income country settings.

**Supplementary Information:**

The online version contains supplementary material available at 10.1186/s12883-026-05017-x.

## Introduction

Stroke, caused by ischemic or hemorrhagic disruption of cerebral blood flow, is the second leading cause of death worldwide [1, 2]. More than half of survivors experience persistent motor, sensory, cognitive, or speech impairments, imposing a substantial burden on individual quality of life and society [2–4]. There are two primary subtypes of stroke— ischemic and hemorrhagic. Although ischemic stroke accounts for the majority of cases, hemorrhagic stroke is associated with higher severity and mortality [2], underscoring the need for subtype-specific analysis [5].

The burden of stroke is particularly severe in low- and middle-income countries (LMICs) where the majority of cases and deaths occur [6, 7]. Among LMICs, countries in Central Asia represent one of the most heavily affected regions worldwide, consistently reporting some of the highest age-standardized stroke mortality and DALY rates globally [8, 9], yet remains markedly underrepresented in stroke research. The Kyrgyz Republic exemplifies this challenge, recording more than 185 stroke-related deaths per 100,000 population annually, with 34.6% of patients dying within one month of onset and nearly half within one year [10, 11]. These rates are substantially higher than those reported in major European countries, highlighting stroke as a serious public health threat in the country [12]. The case-fatality rate in the National Hospital’s Neurology Department No. 2 was 8% in 2024, higher than the hospital’s overall average, reflecting ongoing gaps in acute stroke care.

Predictors of in-hospital case-fatality of stroke have been widely examined. Global studies have identified older age, stroke severity, prior stroke and complications such as pneumonia, renal failure, and hyperglycemia as key risk factors, with additional subtype-specific predictors including atrial fibrillation for ischemic stroke and coma or intraventricular hemorrhage for hemorrhagic stroke [13–16]. Kyrgyz-specific studies have identified similar predictors; however, they have been limited by small sample sizes, separate analyses of stroke subtypes and a narrow focus on clinical variables without incorporating sociodemographic, lifestyle, and care pathway factors [17, 18].

To address these gaps, this study examined all stroke patients admitted to Neurology Department No. 2 of the National Hospital in the Kyrgyz Republic in 2024. Using a multidimensional approach, this study aimed to identify predictors of in-hospital case-fatality for ischemic and hemorrhagic stroke and generate context-specific evidence to improve stroke outcomes in the Kyrgyz Republic and inform strategies for improving stroke outcomes in resource-limited settings.

## Methods

### Conceptual framework

The conceptual framework for this study was based on the variable classification system developed in the INTERSTROKE study by O’Donnell [19]. Although INTERSTROKE was originally designed to identify risk factors for stroke occurrence, many of these factors are also relevant to prognosis and mortality [20]. Thus, its multidimensional structure was considered suitable for in-hospital case-fatality analysis. Based on this framework, we incorporated sociodemographic, lifestyle, medical, and admission-related factors into the analysis.

### Database and sample

This study was designed as a retrospective analysis based on the complete medical records of all patients admitted to Neurology Department No. 2 of the National Hospital of Kyrgyz Republic during 2024 with a primary diagnosis of stroke (ICD-10 codes I60-63). The research data were collected from official admission records which were de-identified by the hospital prior to analysis.

A total of 1,292 patients with confirmed stroke diagnoses were initially identified, comprising 1,057 with ischemic stroke (I63) and 235 with hemorrhagic stroke (I60–I62). Given the differences in pathophysiology, prognosis, and treatment strategies, ischemic and hemorrhagic subtypes were analyzed separately. Patients were excluded if one or more key predictor variables required for the analysis were unavailable. Ultimately, 1,259 patients were eligible for the final analysis, comprising 1,033 with ischemic stroke and 226 with hemorrhagic stroke.

### Definition of variables

#### Outcome variables

The primary outcome was in-hospital case-fatality. Outcome classification was based on the “outcome of inpatient treatment” field in discharge records. Patients were classified as “death” if recorded as “died,” and as “survival” if recorded as “discharged home” or “transferred to another hospital.” This classification reflects patient status at discharge and does not imply post-discharge survival.

#### Explanatory variables

In this study, we included sociodemographic, lifestyle, medical, and admission-related factors to identify factors associated with in-hospital case-fatality among stroke patients.

##### Sociodemographic factors

We included sex, age, area of residence, and social status in sociodemographic factors. Sex was classified as male or female. Stroke incidence is known to increase with age, roughly doubling with each decade after 55 years [21]. Based on this evidence, age was categorized into four groups: <55, 55–64, 65–74, and ≥ 75 years. Area of residence was classified as Bishkek, Chuy, or other provinces. Social status was determined according to the social protection system of the Kyrgyz Republic and categorized as general social protection recipients, pensioners and labor veterans, and others [22]. Pensioners and labor veterans were separated into a distinct category, as this group mainly consists of older individuals with a history of long-term illness or loss of work capacity.

##### Lifestyle factors

Body mass index (BMI), smoking, and alcohol consumption were included in lifestyle factors. BMI was classified according to World Health Organization (WHO) criteria: <25.0 as normal or underweight, 25.0–29.9 as overweight, and ≥ 30.0 as obese [23]. Smoking status and alcohol consumption were each coded as a binary variable (yes/no).

##### Medical factors

The set of medical factors encompassed acute clinical instability, disability status, and prior medical history. Acute clinical instability was assessed using the Modified Early Warning Score (MEWS) which allows for rapid and effective risk stratification even in resource-limited settings [24]. Although MEWS is not a stroke-specific severity scale, prior studies have demonstrated its independent association with in-hospital mortality in acute stroke patients, supporting its use as a pragmatic risk stratification tool in this setting [25]. MEWS is based on five parameters—respiratory rate, heart rate, systolic blood pressure, temperature, and level of consciousness assessed by the AVPU(Alert, Voice,, Pain, Unresponsive) scale. Each parameter is scored from 0 to 3 according to clinical ranges, and higher total scores indicate greater instability [26]. In this study, MEWS was categorized into three groups: low risk (0–2), medium risk (3–4), and high risk (≥ 5). Disability status was classified as present or absent based on the official disability coding system of the Kyrgyz Republic. Past medical history included diabetes, hypertension, hyperlipidemia, myocardial infarction, coronary artery disease, and prior stroke. Each of them was coded as a binary variable (yes/no). However, hypertension was not included in the multivariable regression analyses due to its extremely high prevalence (> 95%) in both ischemic and hemorrhagic stroke groups, resulting in minimal variability. Instead, it is presented as a baseline characteristic.

##### Admission characteristics

Admission-related factors were extracted from medical records and included route of admission and type of admission. The route of admission was classified as self-referral, ambulance, or transfer from another hospital. The type of admission was categorized based on administrative codes recorded at the time of hospital registration, as documented in official admission records: emergency admission within 24 h, emergency admission after 24 h, or non-emergency admission.

### Statistical analysis

All statistical analyses were performed using SAS version 9.4 (SAS Institute, Cary, NC, USA). Descriptive statistics were first calculated for sociodemographic, lifestyle, medical, and admission-related factors and presented as frequencies and percentages. Baseline characteristics were compared using chi-square tests for categorical variables. To further explore sex-specific differences in baseline characteristics, chi-square tests were used to compare sociodemographic, clinical, and admission-related factors between male and female patients, separately within the ischemic and hemorrhagic stroke groups.

To identify independent predictors of in-hospital case-fatality, multivariable logistic regression analysis was conducted. The regression model included sociodemographic, lifestyle, medical, and admission-related variables. Results were reported as adjusted odds ratios (aOR) with two-sided p-values, and statistical significance was set at *p* < 0.05. Multicollinearity among independent variables was assessed using variance inflation factors and none exceeded the threshold of 10.

Additionally, to evaluate the relative contribution of individual physiological parameters, a supplementary analysis was conducted in which the MEWS total score was replaced with its individual components. Temperature and respiratory rate were excluded due to near-zero variance, as the vast majority of patients had scores of 0, precluding reliable estimation.

Model performance was assessed using a stratified nonparametric bootstrap with 2,000 replications to evaluate potential overfitting. Resampling was performed within event and non-event strata, and optimism-corrected AUC with 95% bootstrap percentile confidence intervals was estimated. The impact of missing data was assessed using multiple imputation by chained equations (m = 20). Missing values were imputed using all covariates and the outcome. Results were consistent with the complete-case analysis, supporting the robustness of the findings.

## Results

A total of 1,259 patients were included in the analysis, comprising 1,033 with ischemic stroke and 226 with hemorrhagic stroke. Table 1 presents the baseline characteristics of the study population. During hospitalization, there were 99 in-hospital deaths among ischemic stroke patients (9.6%) and 49 among hemorrhagic stroke patients (21.7%).

**Table 1:** Baseline characteristics of patients with ischemic and hemorrhagic stroke

Category			Ischemic Stroke		Hemorrhagic Stroke		χ2	*p*-value
			*N*	(%)	*N*	(%)		
Total			1033	100.0	226	100.0		
Sociodemographic Factor	Sex	Male	601	58.2	136	60.2	0.30	0.581
		Female	432	41.8	90	39.8		
	Age	< 55 years	170	16.5	72	31.9	37.16	< 0.0001***
		55–64	253	24.5	65	28.8		
		65–74	366	35.4	54	23.9		
		≥ 75 years	244	23.6	35	15.5		
	Area of Residence	Province	531	51.4	113	50.0	3.61	0.1644
		Bishkek	257	24.9	47	20.8		
		Chuy	245	23.7	66	29.2		
	Social Status	Pensioners and labor veterans	500	48.4	84	37.2	11.08	0.0039**
		Social Protection Recipients	236	22.8	71	31.4		
		Others	297	28.8	71	31.4		
Personal and Lifestyle Factor	BMI	Underweight or Normal	361	34.9	56	24.8	6.23	0.0443*
		Overweight	396	38.3	152	67.3		
		Obese	276	26.7	18	8.0		
	Smoking	No	840	81.3	187	82.7	0.25	0.6163
		Yes	193	18.7	39	17.3		
	Alcohol Use	No	916	88.7	189	83.6	4.40	0.036*
		Yes	117	11.3	37	16.4		
Medical Factor	MEWS	Low risk	777	75.2	127	56.2	48.12	< 0.0001***
		Medium risk	210	20.3	65	28.8		
		High risk	46	4.5	34	15.0		
	Disability	No	869	84.1	197	87.2	1.32	0.2499
		Yes	164	15.9	29	12.8		
	DM	No	657	63.6	176	77.9	16.88	< 0.0001***
		Yes	376	36.4	50	22.1		
	Hypertension	No	46	4.5	11	4.9	0.07	0.7862
		Yes	987	95.5	215	95.1		
	Hyperlipidemia	No	540	52.3	127	56.2	1.14	0.2849
		Yes	493	47.7	99	43.8		
	Myocardial Infarction	No	904	87.5	201	88.9	0.35	0.5534
		Yes	129	12.5	25	11.1		
	Coronary artery disease	No	372	36.0	103	45.6	7.22	0.0072**
		Yes	661	64.0	123	54.4		
	History of Stroke	No	683	66.1	162	71.7	2.60	0.1068
		Yes	350	33.9	64	28.3		
Admission Characteristics	Route of Admission	self-referral	388	37.6	56	24.8	24.11	< 0.0001***
		ambulance	509	49.3	152	67.3		
		referred from another hospital	136	13.2	18	8.0		
	Type of Admission	Non-emergency	481	46.6	135	59.7	14.22	0.0008***
		Emergency admission within 24 h	400	38.7	60	26.5		
		Emergency admission after 24 h	152	14.7	31	13.7		

* *p* < 0.05, ** *p* < 0.01, *** *p* < 0.001

### General characteristics of the study participants

In both groups, males were more prevalent. Regarding age distribution, patients younger than 55 years old were more common in the hemorrhagic group, whereas older patients were more frequent in the ischemic group. Approximately half of the patients in both groups resided in Bishkek, where the hospital is located, while fewer than 30% resided in other provinces. Pensioners and labor veterans represented the largest social status category in both groups.

In terms of lifestyle factors, the proportion of overweight patients was highest among those with hemorrhagic stroke (67.3%). In contrast, ischemic stroke patients showed a lower prevalence of overweight (38.3%) but a higher prevalence of obesity (26.7%). Smoking and alcohol consumption were generally low in both groups.

Regarding medical factors, the Modified Early Warning Score (MEWS) revealed clear differences between groups. Low-risk classification was more common in ischemic stroke patients (75.2%) than in hemorrhagic patients (56.2%), whereas medium and high-risk categories were more frequent in the hemorrhagic group.

Hypertension was highly prevalent in both groups, affecting 95.5% of ischemic stroke patients and 95.1% of hemorrhagic stroke patients. Diabetes occurred more frequently in ischemic patients (36.4%) than in hemorrhagic patients (22.1%). By contrast, coronary artery disease was more common in the ischemic group (64.0%) than hemorrhagic group (54.4%). Myocardial infarction showed little difference between groups.

Admission characteristics also differed between groups. Ambulance transport was used more often by hemorrhagic patients (67.3% vs. 49.3%). Non-emergency admission was more common in the hemorrhagic group (59.7%) than in the ischemic group (46.6%). Emergency admission within 24 h was less frequent among hemorrhagic patients (26.5%) compared with ischemic patients (38.7%).

### Factors associated with in-hospital case-fatality among ischemic stroke patients

Table 2 presents the factors associated with in-hospital case-fatality among ischemic stroke patients. Significant predictors included: area of residence, social status, BMI, MEWS, hyperlipidemia, myocardial infarction and route of admission. Patients residing in Bishkek had a significantly higher risk of in-hospital case-fatality compared with those from other provinces (aOR = 4.14, 95% CI 1.72–9.94). In terms of social status, the “Others” group showed a lower risk of case-fatality than pensioners and labor veterans (aOR = 0.36, 95% CI 0.14–0.92), while social protection recipients demonstrated a similar trend but did not reach statistical significance.

**Table 2:** Factors associated with in-hospital case-fatality of ischemic stroke patients

Category			aOR	95% CI (Lower–Upper)		*p*-value
Sociodemographic Factor	Sex	Male	Ref			
		Female	0.54	0.29	1.01	0.0544
	Age	< 55 years	Ref			
		55–64	0.74	0.25	2.19	0.5808
		65–74	0.60	0.20	1.88	0.3834
		≥ 75 years	0.83	0.26	2.71	0.7586
	Area of Residence	Province	Ref			
		Bishkek	4.14	1.72	9.94	0.0015**
		Chuy	1.44	0.51	4.09	0.4974
	Social Status	Pensioners and labor veterans	Ref			
		Social Protection Recipients	0.36	0.13	1.00	0.0509
		Others	0.36	0.14	0.92	0.0325*
Personal and Lifestyle Factor	BMI	Underweight or Normal	Ref			
		Overweight	0.49	0.25	0.98	0.0429*
		Obese	1.14	0.59	2.21	0.6885
	Smoking	No	Ref			
		Yes	0.73	0.32	1.68	0.4598
	Alcohol Use	No	Ref			
		Yes	1.64	0.73	3.72	0.2338
Medical Factor	MEWS	Low risk	Ref			
		Medium risk	12.52	6.54	23.95	< 0.0001***
		High risk	75.08	29.18	193.21	< 0.0001***
	Disability	No	Ref			
		Yes	1.58	0.64	3.91	0.3189
	DM	No	Ref			
		Yes	1.38	0.78	2.43	0.2657
	Hyperlipidemia	No	Ref			
		Yes	0.47	0.26	0.84	0.0102*
	Myocardial Infarction	No	Ref			
		Yes	2.09	1.05	4.18	0.0367*
	Coronary artery disease	No	Ref			
		Yes	1.06	0.53	2.10	0.878
	History of Stroke	No	Ref			
		Yes	1.25	0.70	2.22	0.4507
Admission Characteristics	Route of Admission	self-referral	Ref			
		ambulance	2.84	1.16	6.97	0.0229*
		referred from another hospital	2.02	0.59	6.95	0.265
	Type of Admission	Non-emergency	Ref			
		Emergency admission within 24 h	1.79	0.53	6.04	0.351
		Emergency admission after 24 h	2.97	0.81	10.87	0.1005

* *p* < 0.05, ** *p* < 0.01, *** *p* < 0.001

Overweight patients had a lower case-fatality risk compared with those who were normal or underweight (aOR = 0.49, 95% CI 0.25–0.98). MEWS was a strong predictor, with medium- and high-risk groups showing markedly higher odds of death (aOR = 12.52, 95% CI 6.54–23.95; aOR = 75.08, 95% CI 29.18–193.21, respectively). Hyperlipidemia was associated with lower in-hospital case-fatality (aOR = 0.47, 95% CI 0.26–0.84), whereas myocardial infarction increased the risk of case-fatality (aOR = 2.09, 95% CI 1.05–4.18). Admission by ambulance was also associated with greater case-fatality compared with self-referral (aOR = 2.84, 95% CI 1.16–6.97). Sex, age, diabetes, hypertension, and prior stroke were not significantly associated with in-hospital case-fatality.

Bootstrap validation suggested acceptable model performance, with an optimism-corrected AUC of 0.89 (95% CI: 0.85–0.92). Sensitivity analysis using multiple imputation showed overall similar patterns to the complete-case analysis, although statistical significance changed for some covariates (Supplementary Table 5).

### Factors associated with in-hospital case-fatality among hemorrhagic stroke patients

Table 3 shows that in hemorrhagic stroke, sex, acute clinical instability (MEWS) and myocardial infarction were significantly associated with in-hospital case-fatality. Female patients had nearly a threefold higher risk compared with males (aOR = 2.83, 95% CI 1.09–7.36). MEWS was again a powerful predictor with medium- and high-risk groups showing markedly elevated odds of death relative to the low-risk group (aOR = 7.52, 95% CI 2.23–25.40; aOR = 51.30, 95% CI 12.73–206.73, respectively).

In terms of medical history, myocardial infarction significantly increased case-fatality risk (aOR = 5.94, 95% CI 1.45–24.29). Coronary artery disease and prior stroke were not significant predictors. Pre-hospital factors including route of admission and type of admission were not significantly related to in-hospital case-fatality.

**Table 3:** Factors associated with in-hospital case-fatality of hemorrhagic stroke patients

Category			aOR	95% CI (Lower–Upper)		*p*-value
Sociodemographic Factor	Sex	Male	Ref			
		Female	2.83	1.09	7.36	0.0325*
	Age	< 55 years	Ref			
		55–64	1.61	0.44	5.82	0.4704
		65–74	1.20	0.29	5.01	0.7984
		≥ 75 years	1.62	0.34	7.59	0.5431
	Area of Residence	Province	Ref			
		Bishkek	1.06	0.36	3.15	0.9142
		Chuy	0.58	0.13	2.48	0.459
	Social Status	Pensioners and labor veterans	Ref			
		Social Protection Recipients	2.12	0.52	8.59	0.2922
		Others	2.32	0.60	8.97	0.2219
Personal and Lifestyle Factor	BMI	Underweight or Normal	Ref			
		Overweight	0.61	0.21	1.79	0.3666
		Obese	0.47	0.15	1.47	0.1925
	Smoking	No	Ref			
		Yes	0.44	0.11	1.75	0.2426
	Alcohol Use	No	Ref			
		Yes	1.31	0.36	4.77	0.6806
Medical Factor	MEWS	Low risk	Ref			
		Medium risk	7.52	2.23	25.40	0.0012**
		High risk	51.30	12.73	206.73	< 0.0001***
	Disability	No	Ref			
		Yes	1.03	0.27	3.90	0.9705
	DM	No	Ref			
		Yes	0.90	0.31	2.57	0.8368
	Hyperlipidemia	No	Ref			
		Yes	0.72	0.29	1.80	0.4771
	Myocardial Infarction	No	Ref			
		Yes	5.94	1.45	24.29	0.0132*
	Coronary artery disease	No	Ref			
		Yes	1.43	0.54	3.78	0.4712
	History of Stroke	No	Ref			
		Yes	0.63	0.22	1.77	0.3773
Admission Characteristics	Route of Admission	self-referral	Ref			
		ambulance	1.80	0.35	9.43	0.4842
		referred from another hospital	2.06	0.15	28.31	0.5906
	Type of Admission	Non-emergency	Ref			
		Emergency admission within 24 h	3.98	0.68	23.21	0.1247
		Emergency admission after 24 h	1.29	0.16	10.67	0.8112

* *p* < 0.05, ** *p* < 0.01, *** *p* < 0.001

The internal validation using stratified nonparametric 2,000 bootstrap replicates demonstrated stable model performance. Key predictors retained consistent directions and magnitudes of effects, with optimism corrected AUC of 0.81 (95% CI: 0.76–0.87). Findings from the multiple imputation analysis were comparable to those of the complete-case analysis, with no notable changes in the direction or statistical significance of the associations (Supplementary Table 6).

## Discussion

This study conducted a multidimensional analysis of 1,259 stroke patients admitted to Neurology Department No. 2 of the National Hospital of Kyrgyz Republic in 2024 to identify in-hospital case-fatality factors for ischemic and hemorrhagic subtypes. Across both subtypes, MEWS at admission was the strongest predictor of in-hospital case-fatality. In ischemic stroke, residence in Bishkek, a history of myocardial infarction and ambulance admission were associated with higher case-fatality, while overweight status and hyperlipidemia were inversely associated. For hemorrhagic stroke, female sex and myocardial infarction were associated with increased case-fatality risk.

The cohort consisted of 1,033 patients with ischemic stroke and 226 with hemorrhagic stroke. The in-hospital case-fatality rate was 9.6% for the ischemic group and 21.7% for the hemorrhagic group. This disparity in case-fatality aligns with previous studies showing that hemorrhagic stroke, while less common, has a significantly higher case-fatality rate than ischemic stroke [2, 27, 28]. This finding that hemorrhagic stroke occurred more frequently at younger ages compared with ischemic stroke aligns with prior reports and may reflect differences in underlying pathophysiology [27, 29].

In terms of lifestyle factors, both ischemic and hemorrhagic stroke patients showed high proportions of overweight and obesity. However, the observed rates of alcohol use and smoking in this study were markedly lower than national estimates [10]. This inconsistency could be due to social desirability bias, where patients may have reported more positive lifestyle choices than were accurate during their hospital assessment [30].

For medical factors, hemorrhagic stroke patients were more frequently classified into the medium- and high-risk categories of the MEWS, indicating a greater degree of physiological instability at admission. The higher prevalence of diabetes in ischemic patients aligns with previous findings that metabolic risk factors are more strongly associated with ischemic stroke [27, 31].

In addition, the prevalence of hypertension exceeded 95% in both groups, consistent with prior studies identifying hypertension as the most common and powerful shared risk factor for both ischemic and hemorrhagic stroke [1, 5, 32]. The high prevalence of hypertension in our cohort reflects the broader stroke epidemiology of Central Asia, where high systolic blood pressure is the dominant driver of stroke mortality and disability [8, 9, 33].

National data further indicate that, despite a hypertension prevalence exceeding the global average, rates of diagnosis, treatment, and effective blood pressure control in the Kyrgyz Republic remain very low [34]. Although national hypertension management initiatives have been introduced, evidence suggests that population-level blood pressure control remains insufficient [35]. Together, these findings suggest that the high in-hospital case-fatality observed in this study may represent the downstream consequence of long-standing and inadequately controlled hypertension.

The higher case-fatality observed among patients residing in Bishkek may reflect referral and access patterns related to the hospital’s role as a national tertiary center. Patients from rural areas may be less likely to reach the hospital, particularly in severe conditions, due to geographical barriers and longer transport times. Pre-hospital delays have been associated with worse outcomes [36–38]. However, this interpretation remains speculative, as data on pre-hospital delays were not available. Therefore, the observed association may reflect referral bias rather than a true geographic disparity. Meanwhile, the lower case-fatality risk observed in the ‘Others’ social security code group is likely attributable to this category comprising a significant number of relatively young and economically active patients.

The lower case-fatality risk observed among overweight patients may be interpreted as the ‘obesity paradox’ reported in numerous studies [38–41]. In the context of acute severe illness, a certain level of body fat and muscle mass may serve as an energy reserve, enhancing resilience to physiological stress [42, 43]. Finally, MEWS was identified as the strongest predictor, indicating that physiological instability upon admission is a critical determinant of in-hospital case-fatality in stroke patients [25].

A lower case-fatality risk was observed among patients with hyperlipidemia, a finding that has been reported in several previous studies [44–47]. One possible explanation is that this group may reflect patients with greater prior engagement with the healthcare system, potentially leading to differences in the management of cardiovascular risk factors. However, this association should be interpreted with caution, as unmeasured treatment-related factors were not captured in this dataset.

In contrast, a history of myocardial infarction was associated with increased case-fatality risk, a finding likely attributable to multifactorial mechanisms rather than a single cause. Patients with prior myocardial infarction often have widespread systemic atherosclerosis, which can lead to larger cerebral infarctions and greater neurological severity at onset [48]. In addition, the use of antiplatelet or anticoagulant therapy may limit acute treatment options, further contributing to poorer outcomes [49].

Finally, the elevated case-fatality risk observed among patients admitted via ambulance should be interpreted with caution. Accordingly, ambulance admission is more appropriately interpreted as a proxy for unmeasured severity rather than an independent prognostic factor, although an independent effect cannot be entirely ruled out. Future studies incorporating more detailed severity measures are needed to better evaluate the independent contribution of ambulance admission.

The higher case-fatality observed among female patients with hemorrhagic stroke is unlikely to be explained solely by biological sex differences. In sex-stratified analyses (Supplementary Table 2), age distribution and most comorbidities did not differ significantly between sexes, although a higher proportion of female patients were classified as social protection recipients. However, this finding should be interpreted with caution given the limited sample size. Overall, the observed sex difference in case-fatality may reflect a combination of clinical and contextual factors.

MEWS also emerged as a strong predictor among hemorrhagic patients, with case-fatality risk increasing exponentially across higher score categories. This finding is consistent with prior evidence demonstrating that MEWS at admission is a powerful and independent predictor of case-fatality in acute stroke [25]. Because the initial severity of intracerebral hemorrhage is rapidly reflected in systemic physiological instability, early assessment using MEWS is critically important for the management of hemorrhagic stroke. The association between prior myocardial infarction and increased case-fatality risk is similar to that observed in ischemic stroke, likely reflecting the inability of an already compromised heart to withstand the acute stress of intracerebral hemorrhage [48].

This study provides early real-world evidence on in-hospital stroke case-fatality in the Kyrgyz Republic by distinguishing between ischemic and hemorrhagic subtypes through a multidimensional approach. Furthermore, its comprehensive analysis incorporated not only clinical indicators but also sociodemographic and lifestyle factors. However, as a single-center retrospective study, its findings have limited generalizability and the potential for information and selection bias inherent in medical record-based analysis cannot be excluded. Patients transferred to other facilities were classified as survivors; however, only one such case was identified in our dataset. Therefore, the potential for outcome misclassification is minimal and unlikely to have materially influenced the results.

Furthermore, MEWS was used as a pragmatic proxy for acute clinical instability as stroke-specific scales were not available in medical records. However, it may not fully capture stroke-specific neurological deficits. Accordingly, some aspects of severity may not have been fully accounted for and variables such as ambulance admission may partly reflect this. Therefore, future multi-center cohort studies incorporating standardized neurological assessments are necessary.

## Conclusion

This study provides real-world evidence on in-hospital case-fatality among stroke patients in the Kyrgyz Republic, highlighting substantial differences between ischemic and hemorrhagic subtypes. Across both subtypes, initial clinical instability at admission emerged as the most important determinant of in-hospital case-fatality, indicating the potential importance of early recognition and timely management of acute stroke. In addition, the high prevalence of hypertension in this cohort suggests that inadequate long-term blood pressure control may contribute to stroke burden and outcomes. Overall, these findings highlight the potential importance of early risk stratification and acute stroke management, particularly in resource-limited settings. Further multi-center studies incorporating detailed neurological severity measures would be valuable to refine risk prediction and guide targeted interventions.

## Supplementary Information

Supplementary Material 1.

## Data Availability

The datasets generated and analyzed during the current study are not publicly available due to privacy restrictions but are available from the corresponding author on reasonable request and with permission of the National Hospital of the Kyrgyz Republic.

## Abbreviations

aOR: Adjusted Odds Ratio
AUC: Area Under the Curve
AVPU: Alert, Voice, Pain, Unresponsive
BMI: Body Mass Index
CI: Confidence Interval
DM: Diabetes Mellitus
ICD: International Classification of Diseases
LMICs: Low- and Middle-Income Countries
MEWS: Modified Early Warning Score
Ref: Reference category
SAS: Statistical Analysis System
WHO: World Health Organization
