# Supplementary material for: Predictors of in-hospital case-fatality in patients with stroke at the National Hospital of the Kyrgyz Republic: a retrospective cohort study
Source: BMC Neurol. 2026 May 29;26:459. doi: 10.1186/s12883-026-05017-x (PMC13366716; doi:10.1186/s12883-026-05017-x)
Supplement: Supplementary file 1 — Supplementary Material 1. [file 12883_2026_5017_MOESM1_ESM.docx]

**Supplementary Table**

Supplementary Table 1. Comparison of baseline characteristics between male and female patients with ischemic stroke

| **Category** | | | **Female** | | **Male** | | **χ²** | **p-value** |
| --- | --- | --- | --- | --- | --- | --- | --- | --- |
|  |  |  | **N** | **(%)** | **N** | **(%)** |  |  |
| **Sociodemographic Factor** | Age | < 55 years | 66 | 15.28 | 104 | 17.30 | 48.68 | <.0001*** |
|  |  | 55-64 | 82 | 18.98 | 171 | 28.45 |  |  |
|  |  | 65-74 | 136 | 31.48 | 230 | 38.27 |  |  |
|  |  | ≥75 years | 148 | 34.26 | 96 | 15.97 |  |  |
|  | Area of Residence | Province | 245 | 56.71 | 286 | 47.59 | 9.46 | 0.0088** |
|  |  | Bishkek | 90 | 20.83 | 167 | 27.79 |  |  |
|  |  | Chuy | 97 | 22.45 | 148 | 24.63 |  |  |
|  | Social Status | Pensioners and labor veterans | 74 | 17.13 | 162 | 26.96 | 48.02 | <.0001*** |
|  |  | Social Protection Recipients | 264 | 61.11 | 236 | 39.27 |  |  |
|  |  | Others | 97 | 22.45 | 203 | 33.78 |  |  |
| **Personal and Lifestyle Factor** | BMI | Underweight or Normal | 165 | 38.19 | 196 | 32.61 | 19.67 | <.0001*** |
|  |  | Overweight | 132 | 30.56 | 264 | 43.93 |  |  |
|  |  | Obese | 135 | 31.25 | 141 | 23.46 |  |  |
|  | Smoking | No | 424 | 98.15 | 416 | 69.22 | 138.46 | <.0001*** |
|  |  | Yes | 8 | 1.85 | 185 | 30.78 |  |  |
|  | Alcohol Use | No | 424 | 98.15 | 492 | 81.86 | 66.36 | <.0001*** |
|  |  | Yes | 8 | 1.85 | 109 | 18.14 |  |  |
| **Medical Factor** | MEWS | Low risk | 310 | 71.76 | 467 | 77.70 | 7.40 | 0.0247* |
|  |  | Medium risk | 105 | 24.31 | 105 | 17.47 |  |  |
|  |  | High risk | 17 | 3.94 | 29 | 4.83 |  |  |
|  | Disability | No | 375 | 86.81 | 494 | 82.20 | 4.00 | 0.0456* |
|  |  | Yes | 57 | 13.19 | 107 | 17.80 |  |  |
|  | DM | No | 263 | 60.88 | 394 | 65.56 | 2.38 | 0.1232 |
|  |  | Yes | 169 | 39.12 | 207 | 34.44 |  |  |
|  | Hypertension | No | 17 | 3.94 | 29 | 4.83 | 0.47 | 0.4939 |
|  |  | Yes | 415 | 96.06 | 572 | 95.17 |  |  |
|  | Hyperlipidemia | No | 223 | 51.62 | 317 | 52.75 | 0.13 | 0.721 |
|  |  | Yes | 209 | 48.38 | 284 | 47.25 |  |  |
|  | Myocardial Infarction | No | 374 | 86.57 | 530 | 88.19 | 0.60 | 0.4394 |
|  |  | Yes | 58 | 13.43 | 71 | 11.81 |  |  |
|  | Coronary artery disease | No | 146 | 33.80 | 226 | 37.60 | 1.58 | 0.2086 |
|  |  | Yes | 286 | 66.20 | 375 | 62.40 |  |  |
|  | History of Stroke | No | 300 | 69.44 | 383 | 63.73 | 3.67 | 0.0555 |
|  |  | Yes | 132 | 30.56 | 218 | 36.27 |  |  |
| **Admission Characteristics** | Route of Admission | self-referral | 145 | 33.56 | 243 | 40.43 | 12.05 | 0.0024** |
|  |  | ambulance | 240 | 55.56 | 269 | 44.76 |  |  |
|  |  | referred from another hospital | 47 | 10.88 | 89 | 14.81 |  |  |
|  | Type of Admission | Non-emergency | 226 | 52.31 | 255 | 42.43 | 9.89 | 0.0071** |
|  |  | Emergency admission within 24 hours | 150 | 34.72 | 250 | 41.60 |  |  |
|  |  | Emergency admission after 24 hours | 56 | 12.96 | 96 | 15.97 |  |  |

Note. * p<0.05, ** p<0.01, *** p<0.001

Supplementary Table 2. Comparison of baseline characteristics between male and female patients with hemorrhagic stroke

| **Category** | | | **Female** | | **Male** | | **χ²** | **p-value** |
| --- | --- | --- | --- | --- | --- | --- | --- | --- |
|  |  |  | **N** | **(%)** | **N** | **(%)** |  |  |
| **Sociodemographic Factor** | Age | < 55 years | 24 | 26.67 | 48 | 35.29 | 6.35 | 0.0956 |
|  |  | 55-64 | 23 | 25.56 | 42 | 30.88 |  |  |
|  |  | 65-74 | 23 | 25.56 | 31 | 22.79 |  |  |
|  |  | ≥75 years | 20 | 22.22 | 15 | 11.03 |  |  |
|  | Area of Residence | Province | 47 | 52.22 | 66 | 48.53 | 0.30 | 0.8618 |
|  |  | Bishkek | 18 | 20.00 | 29 | 21.32 |  |  |
|  |  | Chuy | 25 | 27.78 | 41 | 30.15 |  |  |
|  | Social Status | Pensioners and labor veterans | 26 | 28.89 | 45 | 33.09 | 11.98 | 0.0025** |
|  |  | Social Protection Recipients | 45 | 50.00 | 39 | 28.68 |  |  |
|  |  | Others | 19 | 21.11 | 52 | 38.24 |  |  |
| **Personal and Lifestyle Factor** | BMI | Underweight or Normal | 34 | 37.78 | 37 | 27.21 | 2.84 | 0.2416 |
|  |  | Overweight | 28 | 31.11 | 48 | 35.29 |  |  |
|  |  | Obese | 28 | 31.11 | 51 | 37.50 |  |  |
|  | Smoking | No | 86 | 95.56 | 101 | 74.26 | 17.19 | <.0001*** |
|  |  | Yes | 4 | 4.44 | 35 | 25.74 |  |  |
|  | Alcohol Use | No | 84 | 93.33 | 105 | 77.21 | 10.29 | 0.0013** |
|  |  | Yes | 6 | 6.67 | 31 | 22.79 |  |  |
| **Medical Factor** | MEWS | Low risk | 45 | 50.00 | 82 | 60.29 | 2.73 | 0.2558 |
|  |  | Medium risk | 31 | 34.44 | 34 | 25.00 |  |  |
|  |  | High risk | 14 | 15.56 | 20 | 14.71 |  |  |
|  | Disability | No | 83 | 92.22 | 114 | 83.82 | 3.42 | 0.0646 |
|  |  | Yes | 7 | 7.78 | 22 | 16.18 |  |  |
|  | DM | No | 66 | 73.33 | 110 | 80.88 | 1.79 | 0.1808 |
|  |  | Yes | 24 | 26.67 | 26 | 19.12 |  |  |
|  | Hypertension | No | 6 | 6.67 | 5 | 3.68 | 1.05 | 0.3065 |
|  |  | Yes | 84 | 93.33 | 131 | 96.32 |  |  |
|  | Hyperlipidemia | No | 52 | 57.78 | 75 | 55.15 | 0.15 | 0.6964 |
|  |  | Yes | 38 | 42.22 | 61 | 44.85 |  |  |
|  | Myocardial Infarction | No | 78 | 86.67 | 123 | 90.44 | 0.78 | 0.3758 |
|  |  | Yes | 12 | 13.33 | 13 | 9.56 |  |  |
|  | Coronary artery disease | No | 41 | 45.56 | 62 | 45.59 | 0.00 | 0.9961 |
|  |  | Yes | 49 | 54.44 | 74 | 54.41 |  |  |
|  | History of Stroke | No | 63 | 70.00 | 99 | 72.79 | 0.21 | 0.6481 |
|  |  | Yes | 27 | 30.00 | 37 | 27.21 |  |  |
| **Admission Characteristics** | Route of Admission | self-referral | 23 | 25.56 | 33 | 24.26 | 0.05 | 0.975 |
|  |  | ambulance | 60 | 66.67 | 91 | 66.91 |  |  |
|  |  | referred from another hospital | 7 | 7.78 | 11 | 8.09 |  |  |
|  | Type of Admission | Non-emergency | 54 | 60.00 | 81 | 59.56 | 1.05 | 0.5914 |
|  |  | Emergency admission within 24 hours | 26 | 28.89 | 34 | 25.00 |  |  |
|  |  | Emergency admission after 24 hours | 10 | 11.11 | 21 | 15.44 |  |  |

Note. * p<0.05, ** p<0.01, *** p<0.001

Supplementary Table 3. Multivariable logistic regression of individual MEWS components and in-hospital case-fatality in ischemic stroke patients

| **Category** | | | **aOR** | **95% CI (Lower–Upper)** | | **p-value** |
| --- | --- | --- | --- | --- | --- | --- |
| **Sociodemographic Factor** | Sex | Male | ref |  |  |  |
|  |  | Female | 0.56 | 0.29 | 1.06 | 0.0753 |
|  | Age | < 55 years | ref |  |  |  |
|  |  | 55-64 | 0.82 | 0.25 | 2.70 | 0.7405 |
|  |  | 65-74 | 0.55 | 0.16 | 1.91 | 0.3462 |
|  |  | ≥75 years | 0.73 | 0.20 | 2.69 | 0.6366 |
|  | Area of Residence | Province | ref |  |  |  |
|  |  | Bishkek | 3.36 | 1.38 | 8.19 | 0.0077** |
|  |  | Chuy | 1.06 | 0.36 | 3.10 | 0.9232 |
|  | Social Status | Pensioners and labor veterans | ref |  |  |  |
|  |  | Social Protection Recipients | 0.39 | 0.13 | 1.17 | 0.0937 |
|  |  | Others | 0.35 | 0.13 | 0.93 | 0.0347* |
| **Personal and Lifestyle Factor** | BMI | Underweight or Normal | ref |  |  |  |
|  |  | Overweight | 0.65 | 0.32 | 1.31 | 0.2274 |
|  |  | Obese | 1.11 | 0.56 | 2.21 | 0.7706 |
|  | Smoking | No | ref |  |  |  |
|  |  | Yes | 0.95 | 0.40 | 2.27 | 0.9087 |
|  | Alcohol Use | No | ref |  |  |  |
|  |  | Yes | 1.49 | 0.62 | 3.55 | 0.3708 |
| **Medical Factor** | Systolic Blood Pressure | | 1.70 | 1.08 | 2.67 | 0.0208* |
|  | Heart Rate | | 1.35 | 0.68 | 2.67 | 0.3886 |
|  | AVPU | | 6.29 | 4.30 | 9.20 | <.0001*** |
|  | Disability | No | ref |  |  |  |
|  |  | Yes | 1.21 | 0.46 | 3.20 | 0.6965 |
|  | DM | No | ref |  |  |  |
|  |  | Yes | 1.59 | 0.87 | 2.90 | 0.1309 |
|  | Hyperlipidemia | No | ref |  |  |  |
|  |  | Yes | 0.48 | 0.26 | 0.87 | 0.0165* |
|  | Myocardial Infarction | No | ref |  |  |  |
|  |  | Yes | 1.76 | 0.86 | 3.62 | 0.121 |
|  | Coronary artery disease | No | ref |  |  |  |
|  |  | Yes | 1.11 | 0.54 | 2.30 | 0.7786 |
|  | History of Stroke | No | ref |  |  |  |
|  |  | Yes | 1.25 | 0.68 | 2.29 | 0.4797 |
| **Admission Characteristics** | Route of Admission | self-referral | ref |  |  |  |
|  |  | ambulance | 2.21 | 0.87 | 5.62 | 0.0964 |
|  |  | referred from another hospital | 1.80 | 0.48 | 6.79 | 0.3849 |
|  | Type of Admission | Non-emergency | ref |  |  |  |
|  |  | Emergency admission within 24 hours | 1.35 | 0.38 | 4.81 | 0.6474 |
|  |  | Emergency admission after 24  hours | 2.29 | 0.59 | 8.93 | 0.2315 |

Note. * p<0.05, ** p<0.01, *** p<0.001
*Each MEWS component was entered as a continuous variable (per 1-point increase). Scoring criteria were as follows: Systolic Blood Pressure (mmHg): 0 = 101–199; 1 = 81–100 or ≥200; 2 = 71–80; 3 = ≤70. Heart Rate (beats per minute): 0 = 51–100; 1 = 101–110; 2 = ≤40 or 111–129; 3 = ≥130. Level of Consciousness (AVPU): 0 = Alert; 1 = New agitation or confusion; 2 = Responds to voice; 3 = Responds to pain or unresponsive.
**Temperature and respiratory rate were excluded from the analysis due to near-zero variance, as the vast majority of patients presented with scores of 0 (within normal ranges of 36.1–38.0°C and 9–14 breaths per minute, respectively), precluding reliable estimation.

Supplementary Table 4. Multivariable logistic regression of individual MEWS components and in-hospital case-fatality in hemorrhagic stroke patients

| **Category** | | | **aOR** | **95% CI (Lower–Upper)** | | **p-value** |
| --- | --- | --- | --- | --- | --- | --- |
| **Sociodemographic Factor** | Sex | Male | ref |  |  |  |
|  |  | Female | 4.18 | 1.48 | 11.85 | 0.0071** |
|  | Age | < 55 years | ref |  |  |  |
|  |  | 55-64 | 1.52 | 0.38 | 6.15 | 0.5535 |
|  |  | 65-74 | 0.96 | 0.22 | 4.16 | 0.9564 |
|  |  | ≥75 years | 0.99 | 0.19 | 5.26 | 0.9926 |
|  | Area of Residence | Province | ref |  |  |  |
|  |  | Bishkek | 0.97 | 0.30 | 3.08 | 0.9537 |
|  |  | Chuy | 0.59 | 0.13 | 2.76 | 0.5055 |
|  | Social Status | Pensioners and labor veterans | ref |  |  |  |
|  |  | Social Protection Recipients | 1.40 | 0.32 | 6.18 | 0.6591 |
|  |  | Others | 1.66 | 0.37 | 7.47 | 0.5078 |
| **Personal and Lifestyle Factor** | BMI | Underweight or Normal | ref |  |  |  |
|  |  | Overweight | 0.54 | 0.16 | 1.75 | 0.3012 |
|  |  | Obese | 0.59 | 0.19 | 1.84 | 0.3599 |
|  | Smoking | No | ref |  |  |  |
|  |  | Yes | 0.36 | 0.08 | 1.63 | 0.1833 |
|  | Alcohol Use | No | ref |  |  |  |
|  |  | Yes | 2.13 | 0.55 | 8.21 | 0.2741 |
| **Medical Factor** | Systolic Blood Pressure | | 1.64 | 0.96 | 2.80 | 0.0706 |
|  | Heart Rate | | 1.13 | 0.41 | 3.08 | 0.8118 |
|  | AVPU | | 5.98 | 3.07 | 11.67 | <.0001*** |
|  | Disability | No | ref |  |  |  |
|  |  | Yes | 1.22 | 0.29 | 5.11 | 0.7869 |
|  | DM | No | ref |  |  |  |
|  |  | Yes | 1.15 | 0.39 | 3.40 | 0.7992 |
|  | Hyperlipidemia | No | ref |  |  |  |
|  |  | Yes | 1.08 | 0.40 | 2.92 | 0.8844 |
|  | Myocardial Infarction | No | ref |  |  |  |
|  |  | Yes | 5.38 | 1.27 | 22.74 | 0.0221* |
|  | Coronary artery disease | No | ref |  |  |  |
|  |  | Yes | 1.71 | 0.60 | 4.86 | 0.3126 |
|  | History of Stroke | No | ref |  |  |  |
|  |  | Yes | 0.73 | 0.25 | 2.11 | 0.555 |
| **Admission Characteristics** | Route of Admission | self-referral | ref |  |  |  |
|  |  | ambulance | 0.94 | 0.17 | 5.34 | 0.9422 |
|  |  | referred from another hospital | 1.12 | 0.08 | 16.57 | 0.9346 |
|  | Type of Admission | Non-emergency | ref |  |  |  |
|  |  | Emergency admission within 24 hours | 3.62 | 0.58 | 22.80 | 0.1702 |
|  |  | Emergency admission after 24 hours | 0.89 | 0.10 | 8.14 | 0.9172 |

Note. * p<0.05, ** p<0.01, *** p<0.001
*Each MEWS component was entered as a continuous variable (per 1-point increase). Scoring criteria were as follows: Systolic Blood Pressure (mmHg): 0 = 101–199; 1 = 81–100 or ≥200; 2 = 71–80; 3 = ≤70. Heart Rate (beats per minute): 0 = 51–100; 1 = 101–110; 2 = ≤40 or 111–129; 3 = ≥130. Level of Consciousness (AVPU): 0 = Alert; 1 = New agitation or confusion; 2 = Responds to voice; 3 = Responds to pain or unresponsive.
**Temperature and respiratory rate were excluded from the analysis due to near-zero variance, as the vast majority of patients presented with scores of 0 (within normal ranges of 36.1–38.0°C and 9–14 breaths per minute, respectively), precluding reliable estimation.

Supplementary Table 5. Results of multiple imputation sensitivity analysis for predictors of in-hospital case-fatality in ischemic stroke patients

| **Category** | | | **aOR** | **95% CI (Lower–Upper)** | | **p-value** |
| --- | --- | --- | --- | --- | --- | --- |
| **Sociodemographic Factor** | Sex | Male | ref |  |  |  |
|  |  | Female | 0.49 | 0.27 | 0.90 | 0.022* |
|  | Age | < 55 years | ref |  |  |  |
|  |  | 55-64 | 0.66 | 0.23 | 1.87 | 0.4295 |
|  |  | 65-74 | 0.56 | 0.19 | 1.64 | 0.2909 |
|  |  | ≥75 years | 0.80 | 0.26 | 2.51 | 0.7072 |
|  | Area of Residence | Province | ref |  |  |  |
|  |  | Bishkek | 3.52 | 1.54 | 8.04 | 0.0028** |
|  |  | Chuy | 1.20 | 0.44 | 3.26 | 0.7177 |
|  | Social Status | Pensioners and labor veterans | ref |  |  |  |
|  |  | Social Protection Recipients | 0.43 | 0.16 | 1.15 | 0.0925 |
|  |  | Others | 0.37 | 0.14 | 0.94 | 0.0367* |
| **Personal and Lifestyle Factor** | BMI | Underweight or Normal | ref |  |  |  |
|  |  | Overweight | 0.48 | 0.24 | 0.94 | 0.0333* |
|  |  | Obese | 1.12 | 0.59 | 2.14 | 0.7324 |
|  | Smoking | No | ref |  |  |  |
|  |  | Yes | 0.68 | 0.30 | 1.56 | 0.3659 |
|  | Alcohol Use | No | ref |  |  |  |
|  |  | Yes | 1.61 | 0.72 | 3.58 | 0.2473 |
| **Medical Factor** | MEWS | Low risk | ref |  |  |  |
|  |  | Medium risk | 12.91 | 6.78 | 24.57 | <.0001*** |
|  |  | High risk | 83.31 | 32.74 | 212.02 | <.0001*** |
|  | Disability | No | ref |  |  |  |
|  |  | Yes | 1.37 | 0.57 | 3.32 | 0.4869 |
|  | DM | No | ref |  |  |  |
|  |  | Yes | 1.45 | 0.83 | 2.53 | 0.1965 |
|  | Hyperlipidemia | No | ref |  |  |  |
|  |  | Yes | 0.50 | 0.28 | 0.88 | 0.0158* |
|  | Myocardial Infarction | No | ref |  |  |  |
|  |  | Yes | 2.13 | 1.08 | 4.20 | 0.0286* |
|  | Coronary artery disease | No | ref |  |  |  |
|  |  | Yes | 1.17 | 0.59 | 2.31 | 0.6495 |
|  | History of Stroke | No | ref |  |  |  |
|  |  | Yes | 1.18 | 0.67 | 2.09 | 0.5744 |
| **Admission Characteristics** | Route of Admission | self-referral | ref |  |  |  |
|  |  | ambulance | 3.08 | 1.27 | 7.47 | 0.0128* |
|  |  | referred from another hospital | 2.03 | 0.59 | 7.01 | 0.2622 |
|  | Type of Admission | Non-emergency | ref |  |  |  |
|  |  | Emergency admission within 24 hours | 1.79 | 0.53 | 5.99 | 0.3468 |
|  |  | Emergency admission after 24 hours | 3.14 | 0.87 | 11.37 | 0.0815 |

Note. * p<0.05, ** p<0.01, *** p<0.001

Supplementary Table 6. Results of multiple imputation sensitivity analysis for predictors of in-hospital case-fatality in hemorrhagic stroke patients

| **Category** | | | **aOR** | **95% CI (Lower–Upper)** | | **p-value** |
| --- | --- | --- | --- | --- | --- | --- |
| **Sociodemographic Factor** | Sex | Male | ref |  |  |  |
|  |  | Female | 2.62 | 1.04 | 6.57 | 0.0406* |
|  | Age | < 55 years | ref |  |  |  |
|  |  | 55-64 | 1.64 | 0.48 | 5.63 | 0.4303 |
|  |  | 65-74 | 1.06 | 0.28 | 4.09 | 0.9284 |
|  |  | ≥75 years | 1.98 | 0.44 | 8.90 | 0.3716 |
|  | Area of Residence | Province | ref |  |  |  |
|  |  | Bishkek | 0.98 | 0.35 | 2.75 | 0.9621 |
|  |  | Chuy | 0.56 | 0.14 | 2.23 | 0.4113 |
|  | Social Status | Pensioners and labor veterans | ref |  |  |  |
|  |  | Social Protection Recipients | 2.15 | 0.56 | 8.29 | 0.2658 |
|  |  | Others | 2.52 | 0.66 | 9.60 | 0.1752 |
| **Personal and Lifestyle Factor** | BMI | Underweight or Normal | ref |  |  |  |
|  |  | Overweight | 0.62 | 0.22 | 1.76 | 0.3664 |
|  |  | Obese | 0.53 | 0.18 | 1.59 | 0.2601 |
|  | Smoking | No | ref |  |  |  |
|  |  | Yes | 0.52 | 0.14 | 1.90 | 0.3217 |
|  | Alcohol Use | No | ref |  |  |  |
|  |  | Yes | 1.15 | 0.33 | 4.00 | 0.8258 |
| **Medical Factor** | MEWS | Low risk | ref |  |  |  |
|  |  | Medium risk | 6.01 | 1.95 | 18.50 | 0.0018** |
|  |  | High risk | 41.92 | 11.56 | 152.02 | <.0001*** |
|  | Disability | No | ref |  |  |  |
|  |  | Yes | 0.90 | 0.25 | 3.22 | 0.8702 |
|  | DM | No | ref |  |  |  |
|  |  | Yes | 0.81 | 0.29 | 2.25 | 0.6791 |
|  | Hyperlipidemia | No | ref |  |  |  |
|  |  | Yes | 0.63 | 0.26 | 1.52 | 0.301 |
|  | Myocardial Infarction | No | ref |  |  |  |
|  |  | Yes | 6.58 | 1.75 | 24.84 | 0.0054** |
|  | Coronary artery disease | No | ref |  |  |  |
|  |  | Yes | 1.37 | 0.54 | 3.48 | 0.5022 |
|  | History of Stroke | No | ref |  |  |  |
|  |  | Yes | 0.86 | 0.32 | 2.28 | 0.7592 |
| **Admission Characteristics** | Route of Admission | self-referral | ref |  |  |  |
|  |  | ambulance | 2.48 | 0.50 | 12.22 | 0.2654 |
|  |  | referred from another hospital | 1.79 | 0.13 | 23.93 | 0.6615 |
|  | Type of Admission | Non-emergency | ref |  |  |  |
|  |  | Emergency admission within 24 hours | 2.39 | 0.47 | 12.15 | 0.2934 |
|  |  | Emergency admission after 24 hours | 1.08 | 0.15 | 7.54 | 0.9411 |

Note. * p<0.05, ** p<0.01, *** p<0.001
